# Simulation of the Design Performance of Carbon Fiber/Glass Fiber Hybrid-Reinforced Resin Matrix Composite Rotors

**DOI:** 10.3390/polym17121668

**Published:** 2025-06-16

**Authors:** Chong Li, Jiayou Wang, Meng Li, Haoyu Wang, Yiguo Song, Xiangzhe Meng, Ruiliang Liu

**Affiliations:** 1 College of Mechanical and Electrical Engineering, Harbin Engineering University, Harbin 150001, China; songyiguo@hrbeu.edu.cn (Y.S.);; 2 College of Materials Science and Chemical Engineering, Harbin Engineering University, Harbin 150001, China; 17882564891@hrbeu.edu.cn (J.W.); limeng02666@163.com (M.L.); haoyuwang@hrbeu.edu.cn (H.W.); liuruiliang@hrbeu.edu.cn (R.L.)

**Keywords:** composites rotor, hybrid reinforced composite materials, finite element analysis, high-speed rotor

## Abstract

Composite rotors, attributing to their leveraging characteristics of the light weight, high strength, high rigidity, corrosion resistance, and low noise, can significantly reduce the moment of inertia and enhance equipment operational efficiency. Using carbon fiber/glass fiber hybrid-reinforced resin–matrix composites as the rotor base material, the radial stability of a rotor can be effectively increased by regulating the fiber volume content. Meanwhile, the introduction of glass fiber not only enables the transition between the metal hub and composite rim but also optimizes the cost structure of the composite system, overcoming the economic bottleneck of single carbon fiber-reinforced resin–matrix composite rotors. This paper employs the finite element method to analyze a three-dimensional model of a composite rotor, investigating the performance of its metal hub and hybrid-reinforced resin–matrix composite rim. According to the radial stress distribution of the composite rotor during operation, the mixing ratio of carbon fiber/glass fiber is adjusted. The high-speed rotation condition of the composite rotor at 18,000 revolutions per minute is simulated to verify its safety and reliability.

## 1. Introduction

The flywheel energy storage system (FESS) is a device that utilizes the acceleration and deceleration of rotating rotors to convert electrical energy into mechanical energy and vice versa. It offers several advantages, including a long cycle life, cleanliness, minimal environmental impact, rapid charging and discharging capabilities, high power density, and excellent cycle efficiency. Additionally, the FESS can deliver substantial power output in a short time frame. As a result, it is considered an ideal energy storage solution for the future and has been applied in various fields, such as peak shaving, uninterruptible power supply, power grid stabilization, electric vehicles, and satellite power systems. Furthermore, it is well suited for applications that require frequent charging and discharging over multiple cycles [1,2,3,4].

The energy density is a crucial index for evaluating the performance of FESS. The rotating flywheel stores energy in the form of kinetic energy, which depends on the rotational inertia and angular velocity of the flywheel. To maximize the energy storage capacity and energy density of FESSs, it is essential to increase the rotational inertia while limiting the angular velocity of the flywheel rotor. The impact of angular velocity is particularly significant, as the energy stored in the flywheel is proportional to the moment of inertia of the rotor and to the square of the maximum rotational speed. The stress in the rotor increases with the rise in rotational speed, leading to significant vibration. This, in turn, compromises the safety of the system. To enhance cost performance, it is essential to reduce the costs associated with the flywheel. The International Renewable Energy Agency estimates that by 2030, the installation cost of FESSs will be 35% lower than the current range of USD 1500 to 6000 per kilowatt-hour [1]. The high cost per kilowatt-hour of the FESS and the strength of the rotor material are the critical factors that are hindering the development of flywheel energy storage. The flywheel rotor is one of the most vital components of FESS. The key to reducing the cost of FESS and improving the material strength of the flywheel rotor lies in selecting the appropriate rotor material and designing an effective rotor structure [5,6].

FESS is generally categorized into low-speed FESS and high-speed FESS. The components of the flywheel rotor are primarily manufactured using metal and composite materials. The metal rotor is typically employed in low-speed energy storage flywheels and benefits from relatively mature design and processing technologies. The structural design of a metal flywheel rotor is often optimized by altering the rotor’s shape. Gao [7] utilized metal materials to reduce the manufacturing complexity and cost of FESS. A double-hub combined flywheel was designed to decrease the mass while achieving a greater moment of inertia, thereby enhancing energy storage density. The maximum rotational speed reached up to 13,500 rpm. One of the critical challenges with FESS at high rotational speeds is ensuring the material strength and minimizing the vibration of the flywheel rotor. Higher energy storage density can be attained due to the high specific strength, excellent rigidity, and low specific gravity of composite materials. Consequently, composite materials are the preferred choice for high-speed energy storage flywheels. However, the manufacturing process for composite materials is more complex compared to conventional materials. The composite flywheel rotor has consistently been a focal point of research. In addition to the multi-ring structure, designs incorporating hybrid materials, gradient materials, and pretension fiber winding structures have been developed to enhance energy storage density [7,8,9].

At present, most flywheel rotors utilize a metal hub combined with a composite rim structure. High-strength aluminum alloys, such as 7075, are commonly employed for the metal hub. The composite rim typically consists of materials like carbon fiber, glass fiber, and epoxy resin. The winding process is applied to form the glass or carbon fiber rim. Most rotors are designed in a disc or cylindrical shape, as fabricating composite rotors with complex geometries using the winding process is challenging. Flywheel rotors are generally constructed as thin discs with larger diameters, as this configuration provides a greater moment of inertia compared to thicker discs [5]. The radial strength of the composite flywheel rotor formed by the winding method is much lower than its circumferential strength due to anisotropy. A large enough value of the initial radial compressive stress of a composite flywheel rotor can be achieved using the press-fit forming process with interference fit. The initial radial compressive stress of the rotor can effectively offset the radial centrifugal tensile stress at high rotational speed. The maximum radial stress of a high-speed rotating composite flywheel rotor is significantly reduced [5,10]. Chen [11] designed a 200 kW FESS for uninterruptible power supply (UPS) and utilized a composite rotor with an interference fit. The maximum rotational speed achieved was 20,000 r/min. The composite rotor demonstrated safe and reliable operation during high-speed testing. Kim [12] designed and manufactured a composite rotor capable of operating at a speed of 15,000 r/min. A new hybrid composite hub, filament-wound in a dome shape, was proposed to meet the strength and deformation requirements of the rotor. A press-fit forming process with an interference fit was employed to minimize the radial tensile stresses at the shaft–hub and hub–rotor interfaces. Variation in hub stiffness from the inner surface to the outer surface was achieved through material hybridization and the careful selection of the winding angle, ensuring proper contact between the shaft–hub and hub–rotor at the maximum rotational speed.

A hybrid composite is a composite material composed of more kinds of fiber reinforcements in the same resin matrix. It utilizes the performance advantages of each material to achieve complementary advantages through their combination. A hybrid composite has special properties that are not easy to obtain with only a single type of fiber reinforcement. Fiber hybrids are becoming more convenient and controllable methods not only to meet the requirements of design performance but also to reduce the cost of products, increase the quality of products, prolong the service life of products, and improve the economic benefits [13,14,15]. Glass fiber-reinforced polymer (GFRP) composites and carbon fiber-reinforced polymer (CFRP) composites are among the most commonly used composite materials. Carbon fiber is known for its high strength and high modulus; however, it is significantly more expensive than glass fiber. M. Spencer [16] developed glass/carbon fiber hybrid epoxy composite laminates that effectively balanced cost and performance. Tang C.L. [17] designed and manufactured two hybrid composite flywheels consisting of six layers. The radial gradient material was produced by varying the fiber ratio. The hybrid composite flywheel rotor offers advantages such as a smooth radial stress distribution, high structural safety and reliability, and a flexible and feasible hybrid design method.

In summary, this paper establishes a finite element model to verify the feasibility of the rotor design in an energy storage system. The rotor consists of a hybrid-reinforced composite rim made from carbon fiber and glass fiber, along with a metal hub. This study calculates the impact of hybrid-reinforced composite ring layers at various levels on the rotor’s stress and determines the maximum rotational speed of the rotor. Furthermore, the safety of the flywheel rotor is confirmed through modal analysis. The findings presented in this paper serve as a valuable reference for the design of high-speed composite rotors.

## 2. Stress Analysis of a Rotor

A stress analysis to calculate the deformations and strength ratios of a multi-rim rotor can be found in previous publications [18]. However, a summary of the relevant equations is given below. Equation (1) shows the equation governing a rotor rotating at speed *ω* at radius r in the cylindrical coordinate system:

(1) $$\frac{d\sigma_{r}}{dr}+\frac{\sigma_{r}-\sigma_{\theta }}{r}+\rho r\omega^{2}=0$$

where $\sigma_{r}$ is the radial stress; $\sigma_{\theta }$ is the circumferential stress; *r* is the radius of a rotor; ρ is the density; ω is the rotational speed.

The stress–strain relationship can be given as

(2) σ=Qε

where σ is the stress vector; ε is the strain vector; *Q* is the stiffness matrix.

Equation (2) can be rewritten as

(3) σθσzσr=Q11Q12Q13Q21Q22Q23Q31Q32Q33εθεzεr

where $\sigma_{z}$ is the axial stress; $\varepsilon_{r}$ is the radial strain; $\varepsilon_{\theta }$ is the circumferential strain; $\varepsilon_{z}$ is the axial strain. The stiffness matrix Q is given by the material properties as

(4) Q=1Eθ−υθrEr−υθzEz1Er−υrzEzsym.1Ez−1

where $E_{\theta }$ is the longitudinal Young’s modulus; $E_{z}$ and ${\;E}_{r}$ are the transverse Young’s moduli; $\upsilon_{\theta r}$, $\upsilon_{\theta z},\;$ and $\upsilon_{rz}$ are the Poisson’s ratio.

Assuming the plane stress state, the radial and circumferential strains are expressed as

(5) $$\varepsilon_{\theta }=\frac{u_{r}}{r},\;\;\varepsilon_{r}=\frac{{\partial u}_{r}}{\partial r}$$

where *u_r_* is the radial displacement.

Between two adjacent rings of the rim (e.g., *j* and *j + 1*), the compatibility conditions should be satisfied as follows:

(6) $$\sigma_{r_{i}}^{\left(j+1\right)}=\sigma_{r_{0}}^{\left(j\right)},\;{\;u}_{r_{i}}^{\left(j+1\right)}=u_{r_{0}}^{\left(j\right)},\;(j=1,2,3,\;...,\;N\;-1)$$

where r_i_ and r_0_ are the inner and outer radius of the ring, respectively.

## 3. Finite Element Model

### 3.1. Structure of Rotor

The structure, materials, and manufacturing processes of energy storage flywheel rotors are typically chosen to maximize energy density and specific energy. Due to the anisotropy of the composite material, the radial strength (perpendicular to the fiber direction) of the composite rim is significantly lower than its circumferential strength (aligned with the fiber direction). This discrepancy makes the rotor susceptible to radial delamination failure during high-speed rotation.

**Figure 1:**
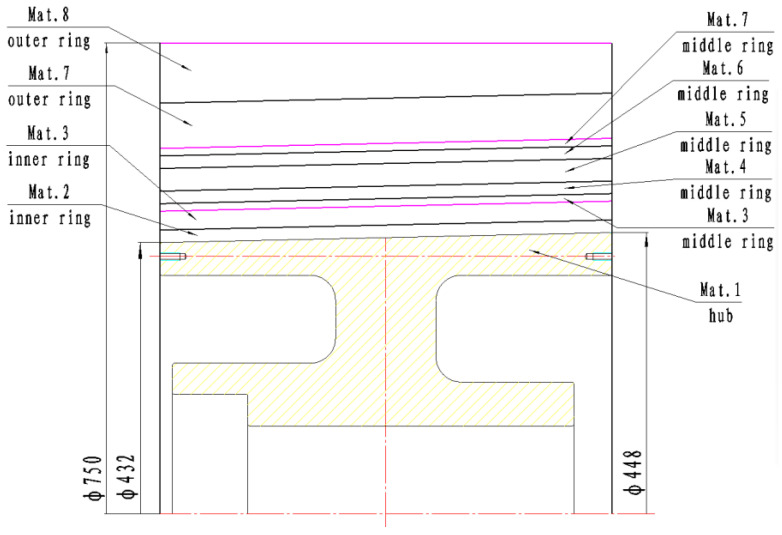
Material distribution of the composite rim of Rotor-B, Rotor-C, and Rotor-E.

In this paper, the composite rim consists of three rings that are fabricated using a winding forming technique and assembled with an interference fit. The impact of the material and thickness distribution of the three rings on the stress state of the rim is analyzed. Five design schemes, labeled Rotor-A, Rotor-B, Rotor-C, Rotor-D and Rotor-E, are proposed, as illustrated in Table 1. For Rotor-B, Rotor-C, and Rotor-E, the material distribution of the composite rim is presented in Figure 1.

### 3.2. Material Properties of the Metal Hub and the Composite Rim

Aluminum alloy 7075, used for the metal hub, was designated as Mat.1 in ANSYS R16.0, and its properties are presented in Table 2.

The glass fiber-reinforced composite (GFRC), carbon fiber-reinforced composite (CFRC), and glass/carbon hybrid composite (GCHC) used for the rim of the flywheel rotor were designated as Mat.2–8 in ANSYS. The ring materials’ parameters exhibit a gradient variation from the inside to the outside, achieved by adjusting the carbon fiber (CF) and glass fiber (GF) contents during the winding process of the composite rings. The material properties of Mat.2 to Mat.8 are presented in Table 3. The longitudinal Young’s modulus, Poisson’s ratio, and density of GCHC with varying hybrid ratios increase linearly with the CF ratio. This behavior can be predicted using the linear mixing law. Additionally, the transverse Young’s modulus of a GCHC can also be estimated based on the mixing law, which has a minimal impact on the stress and deformation results [17,22]. Tang [17] predicted the material constants of GCHC using Equations (7)–(10), based on the mixing law. The mechanical equations of single-layer and multi-layer composite flywheels were established. A six-layer hybrid flywheel was designed and manufactured, and experimental verification was carried out.

The prediction equations for the longitudinal elastic modulus (*E_L_*), transverse elastic modulus (*E_T_*), Poisson’s ratio (*υ*), and density (ρ) are as follows [17]:

(7) $$E_{L}=V_{m}E_{m}+V_{g}E_{g}+V_{c}E_{c}$$

(8) $$\frac{1}{E_{T}}=\frac{V_{m}}{E_{m}}+\frac{V_{g}}{E_{g}}+\frac{V_{c}}{E_{c}}$$

(9) $$\mu_{L}=V_{m}\mu_{m}+V_{g}\mu_{g}+V_{c}\mu_{c}$$

(10) $$\rho =V_{m}\rho_{m}+V_{g}\rho_{g}+V_{c}\rho_{c}$$

where *V* refers the volume fraction, and the subscripts *g*, *c*, and *m* represent GF, CF, and the matrix, respectively.

### 3.3. Model and Mesh of Rotor

**Figure 2:**
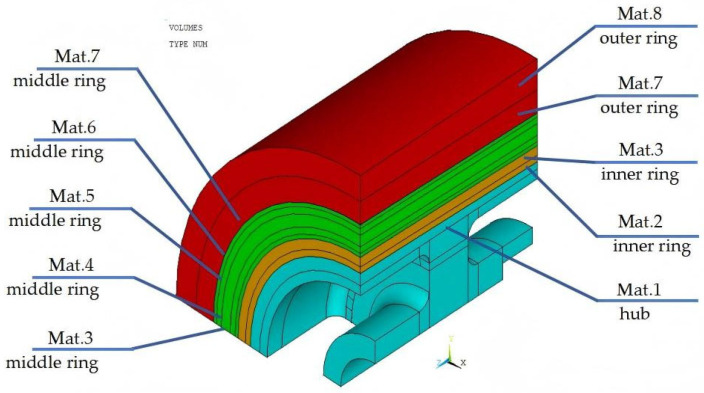
Quarter model of rotor.

**Figure 3:**
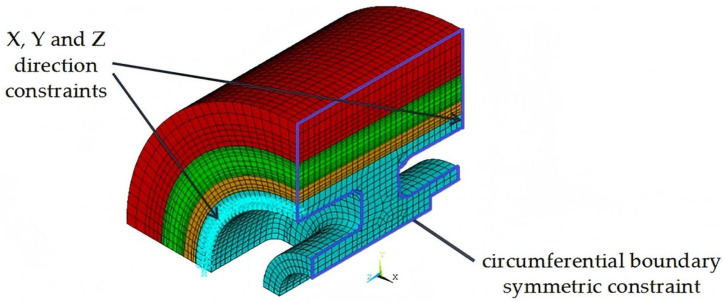
Mesh and boundary conditions of rotor.

A quarter model was built because the rotor is an axisymmetric structure. The cyan part illustrates the metal hub, the yellow part illustrates the inner ring, the green part illustrates the middle ring, and the red part illustrates the outer ring, with the material distribution shown in Figure 2. The 8-node hexahedral elements solid 45 and solid 46 were used to mesh the metal hub and the composite rim, respectively, as shown in Figure 3.

### 3.4. Contact Pair and Boundary Conditions

Contact pairs were established on the contact surfaces between the hub and the inner ring, the inner ring and the middle ring, and the middle ring and the outer ring. Face-to-face contact was utilized to simulate the interference fit of the rotor. The contact surface employed the CONTA174 element, while the target surface used the CONTA170 element. The friction coefficient was set at 0.2, and the effect of temperature on the friction coefficient was not considered.

The level of interference is determined by the offset of the contact surface. If the interference between the rim and the hub, as well as between the rings of the rim, is insufficient, it may lead to the separation or delamination of the rim at high rotational speeds. Conversely, if the interference is excessive, the radial compressive stress on the composite rim’s contact surface may surpass the material’s radial compressive strength, resulting in damage. In this study, the interference amounts between the hub and the inner ring, the inner ring and the middle ring, and the middle ring and the outer ring were all set at 0.3 mm.

To prevent the complete displacement of the model, constraints were applied to specific nodes in ANSYS. Considering the connection between the hub and the shaft via screws, fixed constraints in the X, Y, and Z directions were applied to the nodes located on the circumference where the screws were positioned. A symmetric constraint was imposed on the circumferential boundary, as illustrated in Figure 3. Centrifugal force was applied to the model by utilizing the rotational angular velocity around the Z-axis with the APDL command, in accordance with the actual operating conditions of the flywheel rotor.

## 4. Simulation Results and Discussions

**Figure 4:**
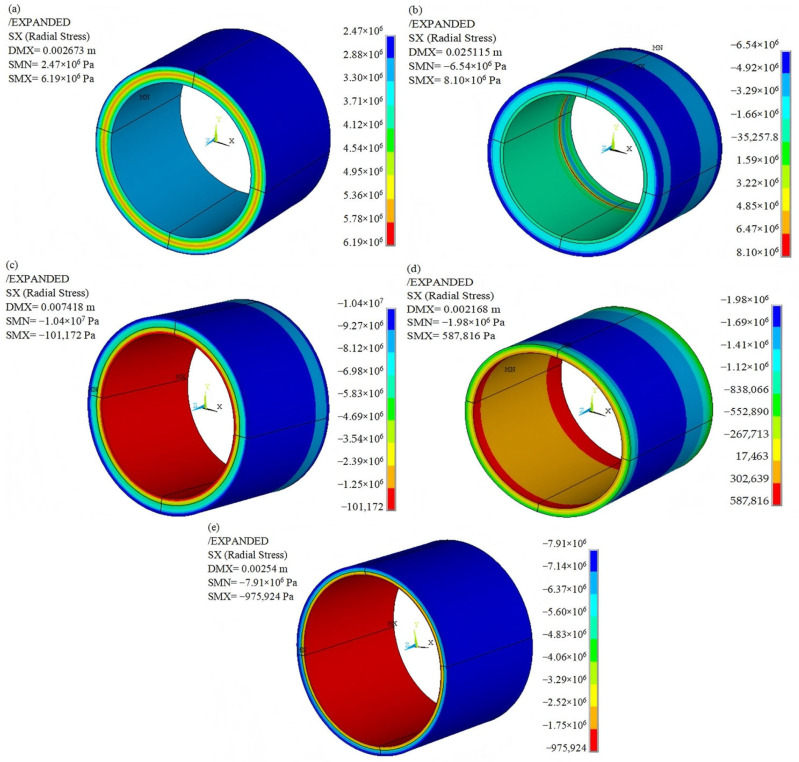
Radial stress distribution of the inner ring: (**a**) Rotor-A; (**b**) Rotor-B; (**c**) Rotor-C; (**d**) Rotor-D; (**e**) Rotor-E. DMX: maximum axial displacement; SMN: minimum radial stress; SMX: maximum radial stress.

The radial strength of the composite ring formed by winding is significantly lower than the circumferential strength, making the rotor with an interference fit susceptible to delamination failure. Consequently, understanding the radial stress distribution of the composite rim is crucial for determining whether a flywheel rotor can operate normally at high rotational speeds. The stress state of the rotor during rotation can be observed in the stress nephograms. Figure 4 illustrates the radial stress nephograms of the inner rings of Rotor-A, Rotor-B, Rotor-C, Rotor-D, and Rotor-E at a speed of 18,000 r/min.

### 4.1. Influence of Hybrid Composite and Thickness of Rings

It is evident that the radial stress values on the inner and outer contact surfaces of the inner ring of Rotor-A are all positive, indicating tensile stress with no compressive stress present, as illustrated in Figure 4a. The rotor may fail due to the separation of the hub and the rim at a speed of 18,000 r/min.

Compared to Rotor-A, both tensile and compressive radial stress zones coexist simultaneously on the contact surface between the hub and the inner ring of Rotor-B, as shown in Figure 4b. The presence of tensile stress on the contact surface indicates that the material or structural design of the rotor should be further optimized to ensure that the radial stress on all contact surfaces is compressive, allowing the rotor to operate normally.

In contrast, both the inner and outer contact surfaces of the inner ring of Rotor-C exhibit only compressive stress, as illustrated in Figure 4c. This design helps prevent the separation of the hub, inner ring, and middle ring, thereby avoiding rotor failure at a speed of 18,000 r/min. The gradient change in the material properties of the hybrid composite improves the radial stress distribution of the rotor. The low specific modulus within the rotor leads to significant deformation, while the high specific modulus on the outer layer results in minimal deformation. Consequently, the deformation of the inner layer is greater than that of the outer layer during rotation. The interaction between the inner and outer layers generates compressive stress, which further mitigates centrifugal tensile stress [17]. To improve the stress distribution of the rotor, the gradient change in the specific modulus of the hybrid composite should be made smoother by adjusting the ratio of the low-modulus GF to the high-modulus CF.

The thickness ratio of the rings from the inside to the outside of Rotor-D is 1:2:3. In comparison to Rotor-A, which has three rings of uniform thickness, the contact surface between the hub and the inner ring of Rotor-D remains within the tensile stress zone. The contact surface between the inner ring and the middle ring of Rotor-D experiences compressive stress, as illustrated in Figure 4d. The increasing thickness of the rings from the inside to the outside improves the radial stress distribution of the rotor to a certain extent.

Analyzing Rotor-C, it can be observed that although both the inner and outer surfaces of the inner ring of the rotor experience compressive stress, the displacement reaches a maximum of 7.418 mm. Consequently, the stress state of Rotor-E, which features a smooth gradient change in the specific modulus and a thickness ratio of rings of 1:2:3 from the inside to the outside, was examined further at a rotational speed of 18,000 r/min. Both the inner and outer contact surfaces of the inner ring of Rotor-E also exhibit compressive stress, as illustrated in Figure 4e. It is evident that the displacement of the inner ring of Rotor-E is 2.54 mm, which is less than that of Rotor-C. Compared to Rotor-C, Rotor-E represents a superior design scheme.

### 4.2. Maximum Rotational Speed Analysis

**Figure 5:**
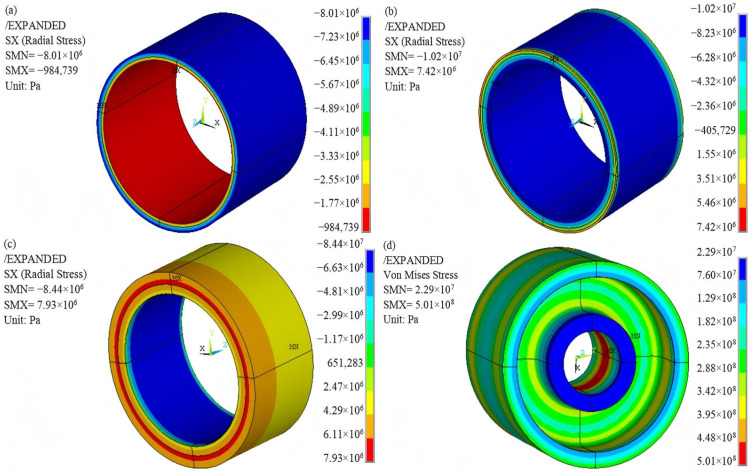
Stress distribution of Rotor-E: (**a**) radial stress distribution of the inner ring; (**b**) radial stress distribution of the middle ring; (**c**) radial stress distribution of the outer ring; (**d**) von Mises equivalent stress distribution of the hub.

The maximum rotational speed of the flywheel rotor was analyzed when further reaching 18,400 r/min. The radial stress distribution of the inner and outer rings of the rim as well as the von Mises equivalent stress distribution of the hub are illustrated in Figure 5 at a speed of 18,400 r/min. It is evident that the contact surfaces between the hub, the inner ring, the middle ring, and the outer ring of the rotor all experience radial compressive stress, as shown in Figure 5a–c.

The tensile strength of the hybrid fiber composite is greater than that of two single fiber composites [22]. The ultimate tensile and compressive strengths in the transverse direction of the glass fiber-reinforced composite (GFRC) are 31 MPa and 118 MPa, respectively [12,17,23]. The radial tensile and compressive stresses of the rim are 7.93 MPa and 10.2 MPa, respectively, which are significantly lower than the ultimate tensile and compressive strengths in the transverse direction of the GFRC. The von Mises equivalent stress distribution is utilized to analyze the operational state of the metal hub. As shown in Figure 5d, the maximum equivalent stress of the hub is 501 MPa, which is lower than the yield stress of 7075 aluminum alloy (546 MPa). This maximum stress occurs at the internal surface of the central hole of the hub. To ensure the safe operation of the rotor, it can be concluded that the rotational speed of Rotor-E should not exceed 18,000 r/min.

An important index to evaluate the performance of FESS, the energy storage density *e*, can be expressed as [24]

(11) $$e=\frac{E_{max}}{m}=\frac{J\omega_{max}^{2}}{2m}$$

where $E_{max}$ is the maximum energy stored by the flywheel; *m* is the mass of the flywheel; *J* is the moment of inertia of the flywheel; $\omega_{max}$ is the maximum angular velocity of the flywheel rim.

(12) $$J=\frac{1}{2}m\left(r_{i}^{2}+r_{e}^{2}\right)=\frac{1}{2}mr_{e}^{2}(\alpha^{2}+1)$$

(13) $$e=\frac{\left(1+\alpha^{2}\right)\upsilon_{max}^{2}}{4}$$

where $r_{i}\;$ is the internal diameters; $r_{e}\;$ is the external diameters; α is the ratio of internal to external diameters; $v_{max}$ the maximum linear velocity of the flywheel rim.

According to Equation (13), the energy storage density of the flywheel rotor is proportional to the square of the rim’s linear velocity. The linear speed of the rotor is constrained by the material’s strength. The maximum linear velocity of a metal flywheel rotor typically ranges from 300 to 500 m/s, as indicated in Table 4 [18,24]. N. Hiroshima [25] employed a three-dimensional CF-reinforced composite material as the rotor material, and the finite element analysis method was utilized to assess the stress distribution of the rotor during high-speed rotation. High-speed spin tests confirmed that the tensile stress generated by the rotor at a speed of 35,900 rpm (526 m/s) led to the rotor’s failure. In contrast, the linear speed of the flywheel rotor analyzed in this paper reaches 706.5 m/s, resulting in a higher energy storage density.

### 4.3. Modal Analysis

A flywheel rotor having an operating frequency range that is close to its natural frequency may lead to structural failure. Therefore, it is essential to conduct a modal analysis during the design of the flywheel rotor to ensure that the natural frequency of the rotor is higher than the operating frequency, thereby preventing mechanical resonance. The modal analysis of the rotor is performed after the static analysis to account for the effects of centrifugal force on the modal characteristics. Once the static analysis is complete, the modal analysis can be initiated by selecting the type, which allows for direct solving without the need to redefine the boundary conditions. The displacement constraint conditions remain the same as those used in the static analysis, and the rotational speed is disregarded in the modal analysis.

The relationship between the rotational speed and the natural frequency can be given as

(14) n=60×f

where *n* is the critical speed; *f* is the natural frequency.

The natural frequencies and critical speeds of Rotor-E are presented in Table 5. At the critical speed, the system may experience resonance or instability, potentially leading to flywheel failure. It is evident that the minimum critical speed of the flywheel is 102,756 r/min, which significantly exceeds the designed speed of 18,000 r/min. Consequently, the flywheel operates outside the resonance region during its functioning.

**Figure 6:**
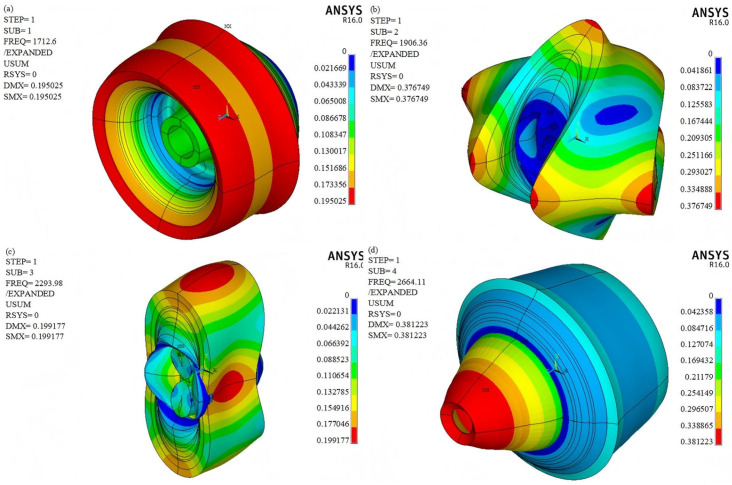
Vibration mode diagrams of Rotor-E: (**a**) First-order vibration mode; (**b**) Second-order vibration mode; (**c**) Third-order vibration mode; (**d**) Fourth-order vibration mode.

The vibration mode diagram of the flywheel rotor illustrates the deformation of the rotor when its rotational speed resonates with the rotor’s natural frequency. These vibration modes depend on the specific design and properties of the rotor. The vibration mode diagram of Rotor-E is shown in Figure 6. This information is valuable for designing and optimizing the flywheel rotor to prevent excessive vibrations.

## 5. Conclusions

In this paper, we discuss the structure and material design of a flywheel rotor featuring a metal hub and a composite rim, utilizing the 3D finite element method. The composite rim consists of three wound rings. The material properties of the hybrid composite gradually vary from the inner to the outer layers by adjusting the GF and CF contents during the winding process of the composite rings. Aluminum alloy 7075 is employed for the metal hub.

The gradient variation in the specific modulus in the hybrid composite can enhance the radial stress distribution of the rotor. A smooth gradient change in the specific modulus of the hybrid composite rim, achieved by adjusting the ratio of GF to CF, represents a more effective approach. Furthermore, increasing the thickness of the rings from the inside to the outside can further optimize the radial stress distribution of the rotor to a certain extent.

Based on the analysis results, the material and structural design of the rotor are finalized. The optimal design of the rim consists of hybrid composite rings with the following thicknesses: the inner ring is 25 mm, the middle ring is 50 mm, and the outer ring is 75 mm, that is, a thickness ratio of 1:2:3 for the inner, middle, and outer rings. The inner ring transitions from 100% GF to 83.3% GF and 16.7% CF, the middle ring transitions from 83.3% GF and 16.7% CF to 16.7% GF and 83.3% CF, and the outer ring transitions from 16.7% GF and 83.3% CF to 100% CF. The hub, inner ring, middle ring, and outer ring are assembled using an interference fit, with an interference amount of 0.3 mm for all components. Through modal analysis, the natural frequencies and mode shapes of the flywheel rotor are established. The rotor avoids the resonance region during operation, indicating that the flywheel is safe to operate at a rotational speed of 18,000 r/min.

## Figures and Tables

**Table 1:** Material and structure design scheme of the composite rim.

Rim Design Scheme	Material of Inner Ring	Thickness of Inner Ring	Material of Middle Ring	Thickness of Middle Ring	Material of Outer Ring	Thickness of Outer Ring
Rotor-A	Mat.2	50 mm	Mat.5	50 mm	Mat.8	50 mm
Rotor-B			Mat.3	6 mm		
	Mat.2	10 mm	Mat.4	10 mm	Mat.7	36 mm
	Mat.3	40 mm	Mat.5	18 mm	Mat.8	14 mm
			Mat.6	10 mm		
			Mat.7	6 mm		
Rotor-C			Mat.3	6 mm		
	Mat.2	20 mm	Mat.4	10 mm	Mat.7	24 mm
	Mat.3	30 mm	Mat.5	18 mm	Mat.8	26 mm
			Mat.6	10 mm		
			Mat.7	6 mm		
Rotor-D	Mat.2	25 mm	Mat.5	50 mm	Mat.8	75 mm
Rotor-E			Mat.3	6 mm		
	Mat.2	10 mm	Mat.4	10 mm	Mat.7	36 mm
	Mat.3	15 mm	Mat.5	18 mm	Mat.8	39 mm
			Mat.6	10 mm		
			Mat.7	6 mm		

**Table 2:** Material properties of the metal hub [19,20,21].

Material Number	Material	Density (kg/m^3^)	Young’s Modulus (GPa)	Poisson’s Ratio	Yield Stress (MPa)
Mat.1	Aluminum alloy 7075	2800	72.5	0.3	546

**Table 3:** Material properties of the composite rim [12,17,23].

Material Number	Material	Longitudinal * Modulus (GPa)	Transversal * Modulus (GPa)	Poisson’s Ratio	Density (kg/m^3^)
Mat.2	GFRC	38.6	8.27	0.26	1800
Mat.3	83.3% GF + 16.7% CF	58	8.4	0.26	1767
Mat.4	66.7% GF + 33.3% CF	77.4	8.5	0.26	1733
Mat.5	50% GF + 50% CF	96.8	8.6	0.26	1700
Mat.6	33.3% GF + 66.7% CF	116.2	8.7	0.26	1667
Mat.7	16.7% GF + 83.3% CF	135.6	8.9	0.26	1633
Mat.8	CFRC	155	9	0.26	1600

* Longitudinal = fiber direction; transversal = vertical to the fiber direction.

**Table 4:** Comparison of metal and hybrid composite flywheel rotors [18,24,25].

Material	Outer Radius (mm)	Rotation Speed (r/min)	Linear Velocity (m/s)
Al 7075 T6	200	20,156	421.9
Steel 18Ni 300	200	15,460	323.6
Stainless steel 455	200	22,087	462.3
Al 6061 T6 kevlar49 epoxy	200	17,006	356
3D CF-reinforced composite	140	35,900	526
Hybrid composite (Rotor-E)	375	18,000	706.5

**Table 5:** First 10th-order natural frequencies and critical speeds of the composite rotor.

Set	Natural Frequency, *f* (Hz)	Critical Speed, *n* (r/min)
1	1712.6	102,756
2	1906.4	114,384
3	2294.0	137,640
4	2664.1	159,846
5	3255.6	375,336
6	3401.2	204,072
7	3483.9	209,034
8	3751.2	225,072
9	3979.6	238,776
10	4030.0	241,800

## Data Availability

The data presented in this study are available upon request from the corresponding author. The data are not publicly available as the data are part of an ongoing study.

## Abbreviations

The following abbreviations are used in this manuscript:

FESS	Flywheel Energy Storage System
GFRP	Glass Fiber-Reinforced Polymer
CFRP	Carbon Fiber-Reinforced Polymer
GFRC	Glass Fiber-Reinforced Composite
GCHC	Glass/Carbon Hybrid Composite
GF	Glass Fiber
CF	Carbon Fiber
